# Common psychiatric and metabolic comorbidity of adult attention-deficit/hyperactivity disorder: A population-based cross-sectional study

**DOI:** 10.1371/journal.pone.0204516

**Published:** 2018-09-26

**Authors:** Qi Chen, Catharina A. Hartman, Jan Haavik, Jaanus Harro, Kari Klungsøyr, Tor-Arne Hegvik, Rob Wanders, Cæcilie Ottosen, Søren Dalsgaard, Stephen V. Faraone, Henrik Larsson

**Affiliations:** 1 Department of Medical Epidemiology and Biostatistics, Karolinska Institutet, Stockholm, Sweden; 2 Department of Psychiatry, University of Groningen, University Medical Center Groningen, Groningen, The Netherlands; 3 Division of Psychiatry, Haukeland University Hospital, Bergen, Norway; 4 K.G. Jebsen Centre for Neuropsychiatric Disorders, Department of Biomedicine, University of Bergen, Bergen, Norway; 5 Division of Neuropsychopharmacology, Department of Psychology, University of Tartu, Tartu, Estonia; 6 Department of Global Public Health and Primary Care, University of Bergen, Bergen, Norway; 7 Division of Mental and Physical Health, Norwegian Institute of Public Health, Bergen, Norway; 8 National Centre for Register-based Research, Aarhus University, Aarhus, Denmark; 9 The Lundbeck Foundation Initiative for Integrative Psychiatric Research, iPSYCH, Aarhus, Denmark; 10 Department for Child and Adolescent Psychiatry, Hospital of Telemark, Kragerø, Norway; 11 Departments of Psychiatry and Neuroscience and Physiology, SUNY Upstate Medical University, New York, United States of America; 12 School of Medical Sciences, Örebro University, Örebro, Sweden; Chiba Daigaku, JAPAN

## Abstract

Attention-deficit/hyperactivity disorder (ADHD) is often comorbid with other psychiatric conditions in adults. Yet, less is known about its relationship with common metabolic disorders and how sex and ageing affect the overall comorbidity patterns of adult ADHD. We aimed to examine associations of adult ADHD with several common psychiatric and metabolic conditions. Through the linkage of multiple Swedish national registers, 5,551,807 adults aged 18 to 64 years and living in Sweden on December 31, 2013 were identified and assessed for clinical diagnoses of adult ADHD, substance use disorder (SUD), depression, bipolar disorder, anxiety, type 2 diabetes mellitus (T2DM), and hypertension. Logistic regression models and regression standardization method were employed to obtain estimates of prevalence, prevalence difference (PD), and prevalence ratio (PR). All comorbid conditions of interest were more prevalent in adults with ADHD (3.90% to 44.65%) than in those without (0.72% to 4.89%), with the estimated PRs being over nine for psychiatric conditions (p < 0.001) and around two for metabolic conditions (p < 0.001). Sex differences in the prevalence of comorbidities were observed among adults with ADHD. Effect modification by sex was detected on the additive scale and/or multiplicative scale for the associations of adult ADHD with all comorbidities. ADHD remained associated with all comorbidities in older adults aged 50 to 64 when all conditions were assessed from age 50 onwards. The comorbidity patterns of adult ADHD underscore the severity and clinical complexity of the disorder. Clinicians should remain vigilant for a wide range of psychiatric and metabolic problems in ADHD affected adults of all ages and both sexes.

## Introduction

Attention-deficit/hyperactivity disorder (ADHD) is a common childhood-onset neuropsychiatric disorder that often persists into later life [1]. The prevalence of ADHD has been estimated to be 2.5–5% in adults [2, 3].

Clinical studies have documented that adults with ADHD tend to suffer from psychiatric disorders such as substance use disorder (SUD), depression, bipolar disorder, and anxiety [4, 5]. Most of these studies, however, were based on small samples and non-standard assessment approaches for either ADHD or the psychiatric conditions. Moreover, at least three important questions remain to be addressed. First, it is unclear whether clinically diagnosed adult ADHD is associated with common metabolic conditions, such as type 2 diabetes mellitus (T2DM) and hypertension. Despite population-based evidence for an association between ADHD and T2DM in children and adolescents [6], conclusions in adults remain uncertain [7]. With regard to comorbid hypertension, the largest study to date reported an association between three or more self-reported hyperactive-impulsive symptoms and hypertension in young adults [8]. Clinical assessment of adult ADHD was, however, unavailable in the study. Second, prior studies on sex differences in the comorbidity patterns of adult ADHD were mainly performed in small clinical samples [9, 10], providing no evidence supporting sex differences in the patterns. To ensure sufficient statistical power for such investigation, a large sample of adults is needed. Third, although ADHD symptoms seem to persist even into senior age [11, 12] and remain associated with SUD, depression, and anxiety [13, 14], comorbidity rates in elderly patients with ADHD have not been thoroughly investigated in a large representative sample.

In this population-based cross-sectional study, we sought to provide a broader perspective on the comorbidity patterns of adult ADHD by estimating associations of adult ADHD with several common psychiatric and metabolic conditions, using data from the Swedish national registers. Specifically, we aimed to replicate previously reported associations of adult ADHD with psychiatric conditions, including SUD, depression, bipolar disorder, and anxiety, and explore associations with metabolic conditions, in particular T2DM and hypertension. We then assessed sex differences in these associations, and examined the comorbidity patterns of adult ADHD in individuals aged 50 to 64 years.

## Materials and methods

### Data sources

All residents in Sweden are assigned a unique personal identity number that allows for large-scale linkage across the Swedish national registers [15]. The Total Population Register was established in 1968 and contains information on births, deaths, and migrations [16]. The Swedish National Patient Register covers somatic inpatient care since 1968 and psychiatric inpatient care since 1973, with complete national coverage achieved since 1987 (1973: 86%, 1986:98%) [17]. Since 2001 the register also covers approximately 80% of outpatient visits including day surgery and psychiatric care from both private and public caregivers. Primary care (e.g. general practitioners) is not yet covered in the register. The study was approved by the Regional Ethical Review Board in Stockholm, Sweden (registration number 2013/862-31/5).

### Study population

The study population consisted of 5,551,807 adults aged 18 to 64 years and living in Sweden on December 31, 2013, who were identified from the Swedish Total Population Register. A subgroup of 1,665,938 adults aged 50 to 64 were selected for exploring the comorbidity patterns of adult ADHD in older adults.

### Ascertainment of adult ADHD and comorbidities

Data on diagnoses of adult ADHD and comorbidities under study were retrieved from the Swedish National Patient Register [17]. Ascertainment of adult ADHD was based on presence of at least one diagnosis of ADHD (ICD-9: 314; ICD-10: F90) between age 18 and 64. Similarly, ascertainment of each comorbid condition was based on presence of at least one diagnosis of the condition between age 18 and 64. In the current study, we considered four psychiatric conditions (SUD, depression, bipolar disorder, and anxiety) and two metabolic conditions (T2DM and hypertension). ICD codes for all conditions can be found in S1 Table.

### Statistical analysis

First, we estimated prevalence of each comorbid condition in adults with and without ADHD, as well as the corresponding prevalence ratio (PR), using logistic regression models and regression standardization method [18]. The models were adjusted for two categorical covariates, sex and age in years. For T2DM and hypertension, we conducted additional analyses restricted to individuals without SUD, depression, bipolar disorder, and anxiety.

Second, we tested the presence of effect measure modification (hereinafter referred to as effect modification) by sex on the observed associations. We began with estimating sex-stratified PD for each comorbid condition, comparing adults with ADHD to those without. PD thus reflects the absolute prevalence increase of a comorbid condition associated with ADHD. To assess effect modification by sex on the additive PD scale for the associations, we calculated difference in PD between females and males as

$$Difference\;in\;PD=(P_{11}-P_{00})-[\left(P_{10}-P_{00})+(P_{01}-P_{00}\right)]=(P_{11}-P_{10})-(P_{01}-P_{00})$$

Where *P*_*ij*_ is the prevalence of a comorbid condition in the stratum of sex *i* and adult ADHD status *j*, with *i* = 0 denoting male, *i* = 1 denoting female, *j* = 0 denoting absence of adult ADHD, *j* = 1 denoting presence of adult ADHD. Essentially, difference in PD reflects whether the joint effect of both female sex and adult ADHD on the prevalence difference scale is different (difference in PD ≠ 0) from the sum of the effects of female sex alone and adult ADHD alone. Difference in PD = 0 indicates no effect modification by sex on the additive PD scale. Next, we estimated sex-stratified PR for each comorbid condition, comparing adults with ADHD to those without. To evaluate whether the associations were modified by sex on the multiplicative PR scale, we calculated ratio of PR in females to PR in males for the comorbid condition as

$$Ratio\;of\;PRs=(P_{11}/P_{00})/[\left(P_{10}/P_{00})\times (P_{01}/P_{00}\right)]=\left(P_{11}/P_{10}\right)/\left(P_{01}/P_{00}\right)$$

Ratio of PRs reflects whether the joint effect of female sex and adult ADHD on the PR scale is different (ratio of PRs ≠ 1) from the product of the effects of female sex alone and adult ADHD alone. Ratio of PRs = 1 indicates no effect modification by sex on the multiplicative PR scale. Despite assessment of effect modification by sex on two selected scales in the current study, other scales might be of biological importance as well. For an association of interest, effect modification by sex could be present on any scale but not necessarily all. Nevertheless, assessing additive effect modification is considered important for identifying a target group for intervention and thus more relevant to public health decision-making [19]. Logistic regression models and regression standardization method [18] were used to obtain estimates of PD, difference in PD, PR, ratio of PRs, and 95% CI. The analyses were adjusted for age in years.

Finally, we explored the associations among older adults aged 50 to 64, with diagnoses of all conditions being assessed from age 50 onwards. The analyses were adjusted for sex and age in years.

Robust standard errors were calculated to construct 95% confidence intervals (CIs), accounting for data clustering within the same families. The significance level for all tests was set at p < .05, two-tailed. SAS software version 9.4 (SAS Institute Inc., Cary, NC) was used for data mining. R software version 3.4.1 [20] was used for statistical analyses.

## Results

The study population comprised 5,551,807 adults (49.19% females) aged 18 to 64 years (Mean [SD] age: 40.55 [13.49] years) and living in Sweden on December 31, 2013, of whom 61,129 (1.10%) were diagnosed with ADHD at some point in their adult life. More descriptive information on the distribution of sex, age, and number of psychiatric and metabolic conditions by adult ADHD status is shown in Table 1.

**Table 1:** Descriptive characteristics of study population.

Characteristic	With adult ADHD (N = 61,129)		Without adult ADHD (N = 5,490,678)	
	N	%	N	%
**Sex**				
Female	26,737	43.74	2,704,211	49.25
Male	34,392	56.26	2,786,467	50.75
**Age (years)**				
18–24	20,854	34.11	861,666	15.69
25–34	18,068	29.56	1,159,164	21.11
35–44	11,519	18.84	1,169,103	21.29
45–54	8007	13.10	1,207,419	21.99
55–64	2681	4.39	1,093,326	19.91
**Number of psychiatric conditions other than ADHD**				
0	20,196	33.04	4,940,576	89.98
1	17,583	28.76	380,791	6.94
2	14,288	23.37	128,436	2.34
3	7466	12.21	36,258	0.66
4	1596	2.61	4617	0.08
**Number of metabolic conditions**				
0	58,398	95.53	5,199,707	94.70
1	2,346	3.84	244,507	4.45
2	385	0.63	46,464	0.85
**Affected by both psychiatric and metabolic conditions other than ADHD**				
No	58,828	96.24	5,430,337	98.90
Yes	2301	3.76	60,341	1.10

### Associations of adult ADHD with psychiatric and metabolic conditions

The prevalence estimates of SUD (35.12%), depression (42.28%), and anxiety (44.65%) were more than nine times higher in adults with ADHD than in those without (Table 2). Bipolar disorder, though relatively rare in the general adult population (0.72%), was highly prevalent in adults with ADHD (14.29%), giving a PR of 19.96. Adults with ADHD showed an increased prevalence of T2DM (3.90%) and hypertension (8.51%) compared to those without ADHD, with PRs being 2.41 and 1.90, respectively (Table 2). All associations were still statistically significant when the analyses were stratified by sex (Table 3). Furthermore, when the analyses were restricted to individuals without SUD, depression, bipolar disorder, and anxiety, adult ADHD remained significantly associated with T2MD (PR: 2.11, 95% CI: 1.79–2.42) and hypertension (PR: 1.72, 95% CI: 1.56–1.89).

**Table 2:** Associations between adult ADHD and comorbidities in adults aged 18 to 64^a^.

Comorbidity	With adult ADHD (N = 61,129)			Without adult ADHD (N = 5,490,678)			PR	95% CI
	N	Prevalence, %	95% CI, %	N	Prevalence, %	95% CI, %		
**SUD**	19,444	35.12	34.73–35.51	198,312	3.61	3.59–3.62	9.74	9.62–9.86
**Depression**	22,998	42.28	41.88–42.67	258,175	4.69	4.68–4.71	9.01	8.92–9.10
**Bipolar Disorder**	7123	14.29	13.98–14.60	39,403	0.72	0.71–0.72	19.96	19.48–20.43
**Anxiety**	25,376	44.65	44.25–45.05	269,015	4.89	4.88–4.91	9.12	9.04–9.21
**T2DM**	1044	3.90	3.68–4.13	89,565	1.62	1.61–1.63	2.41	2.27–2.55
**Hypertension**	2072	8.51	8.19–8.83	247,870	4.48	4.47–4.50	1.90	1.83–1.97

^a^ Clinical diagnoses of adult ADHD and comorbid conditions were assessed between age 18 and 64. Estimates of prevalence and PR were adjusted for sex and age in years.

SUD: substance use disorder; T2DM: Type 2 diabetes mellitus; PR: prevalence ratio; CI: confidence interval

**Table 3:** Modifying effects of sex on associations between ADHD and comorbidities in adults aged 18 to 64^a^.

Comorbidity	PD, %	95% CI, %	Additive effect modification by sex			PR	95% CI	Multiplicative effect modification by sex		
			Difference in PD^b^, %	95% CI, %	P value			Ratio of PRs^c^	95% CI	P value
**SUD**										
Female	28.09	27.52–28.66	-6.96	-7.72 –-6.19	<0.001	11.06	10.84–11.28	1.23	1.20–1.26	<0.001
Male	35.05	34.52–35.57				8.97	8.84–9.10			
**Depression**										
Female	43.40	42.80–44.00	11.35	10.57–12.14	<0.001	8.39	8.28–8.50	0.84	0.82–0.85	<0.001
Male	32.05	31.53–32.56				10.03	9.87–10.18			
**Bipolar Disorder**										
Female	18.04	17.53–18.55	8.61	8.00–9.22	<0.001	20.78	20.16–21.40	1.10	1.04–1.15	<0.001
Male	9.43	9.07–9.78				18.94	18.21–19.67			
**Anxiety**										
Female	46.55	45.95–47.15	13.17	12.39–13.96	<0.001	8.52	8.42–8.63	0.84	0.82–0.85	<0.001
Male	33.38	32.86–33.90				10.17	10.02–10.32			
**T2DM**										
Female	2.30	1.97–2.63	-0.06	-0.52–0.40	0.792	2.79	2.53–3.05	1.27	1.12–1.42	0.001
Male	2.36	2.04–2.68				2.20	2.04–2.37			
**Hypertension**										
Female	3.40	2.95–3.86	-1.19	-1.83 –-0.55	<0.001	1.85	1.74–1.97	0.96	0.89–1.04	0.361
Male	4.59	4.14–5.04				1.92	1.83–2.01			

^a^ Clinical diagnoses of adult ADHD and comorbid conditions were diagnosed between age 18 and 64. All estimates were adjusted for age in years

^b^ *Difference in PD* = *PD*_*female*_ − *PD*_*male*_

^c^ *Ratio of PRs* = *PR*_*female*_/*PR*_*male*_

SUD: substance use disorder; T2DM: Type 2 diabetes mellitus; PD: prevalence difference; PR: prevalence ratio; CI: confidence interval

### Sex differences in prevalence of comorbidities among adults with ADHD

**Fig 1:**
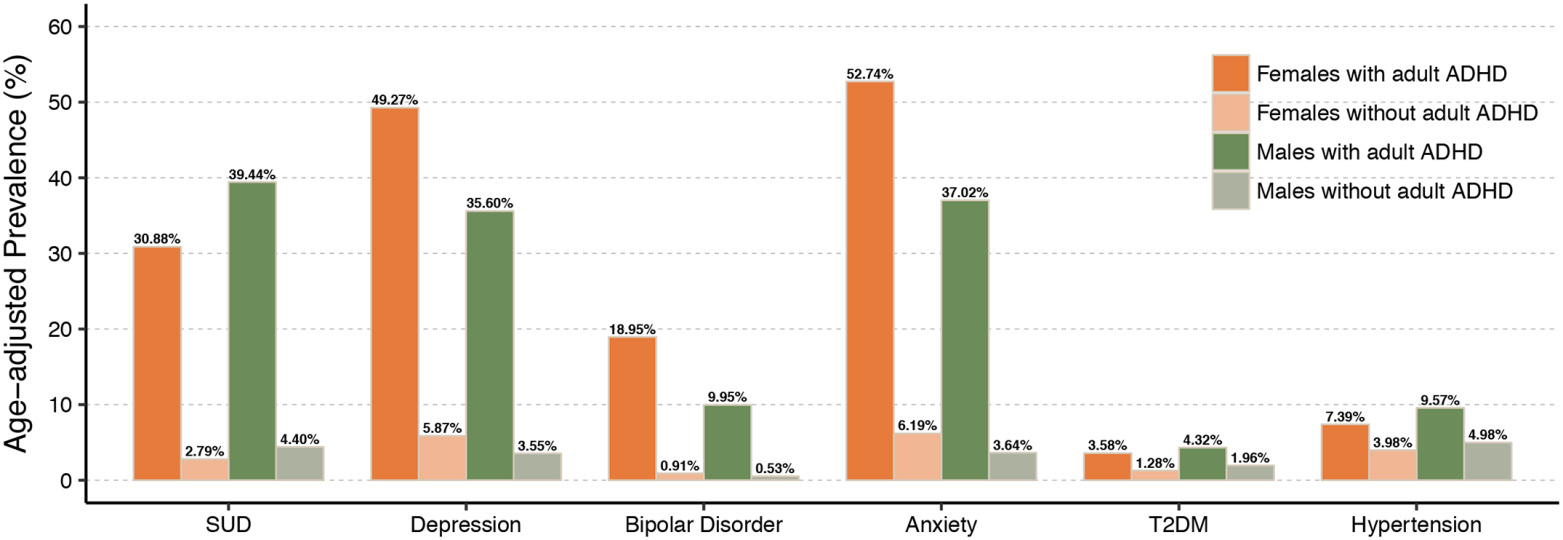
Age-adjusted prevalence estimates of psychiatric and metabolic conditions by sex and adult ADHD status in 5,551,807 individuals aged 18 to 64. Adult ADHD and comorbid conditions were assessed between age 18 and 64.

Among adults with ADHD, depression, bipolar disorder, and anxiety were more prevalent in females than in males, whereas SUD, T2DM, and hypertension were more prevalent in males than in females (Fig 1). The most common comorbidity among females with adult ADHD was anxiety, followed by depression, SUD, bipolar disorder, hypertension, and T2DM. The most common comorbidity among males with adult ADHD was SUD, followed by anxiety, depression, bipolar disorder, hypertension, and T2DM (Fig 1).

### Additive effect modification by sex

We observed additive modifying effects of sex on the associations of adult ADHD with all comorbidities except T2DM (Table 3). For depression, bipolar disorder, and anxiety, adult ADHD was associated with larger absolute prevalence increase (measured by PD) in females than in males (Table 3). For SUD and hypertension, adult ADHD was associated with larger absolute prevalence increase males than in females (Table 3).

### Multiplicative effect modification by sex

Multiplicative effect modification by sex was detected for the associations of adult ADHD with all comorbidities except hypertension (Table 3). Females showed more elevated PRs for SUD, bipolar disorder, and T2DM compared to males, while males had higher PRs for depression and anxiety than females (Table 3).

### Comorbidity of ADHD in older adults

When diagnoses of adult ADHD and comorbidities under study were assessed from age 50 onwards, the prevalence of ADHD was 0.29% in adults aged 50 to 64. The associations of adult ADHD with all comorbidities remained statistically significant (Table 4).

**Table 4:** Associations between adult ADHD and comorbidities in adults aged 50 to 64^a^.

Comorbidity	With adult ADHD (N = 4,864)			Without adult ADHD (N = 1,661,074)			PR	95% CI
	N	Prevalence, %	95% CI, %	N	Prevalence, %	95% CI, %		
**SUD**	1672	35.95	34.61–37.30	50,006	3.01	2.98–3.04	11.95	11.49–12.40
**Depression**	1757	38.79	37.43–40.16	53,593	3.23	3.20–3.25	12.03	11.59–12.46
**Bipolar Disorder**	712	15.43	14.39–16.46	10,802	0.65	0.64–0.66	23.72	22.06–25.38
**Anxiety**	1741	38.12	36.74–39.49	49,909	3.00	2.98–3.03	12.69	12.22–13.16
**T2DM**	253	6.10	5.38–6.82	58,958	3.55	3.52–3.58	1.72	1.52–1.92
**Hypertension**	674	16.65	15.54–17.76	170,191	10.24	10.19–10.29	1.63	1.52–1.73

^a^ Clinical diagnoses of adult ADHD and comorbid conditions were assessed between age 50 and 64. Estimates of prevalence and PR were adjusted for sex and age in years.

SUD: substance use disorder; T2DM: Type 2 diabetes mellitus; PR: prevalence ratio; CI: confidence interval

## Discussion

This large population-based investigation of common psychiatric and metabolic comorbidity of adult ADHD generated three main findings. First, SUD, depression, bipolar disorder, anxiety, T2DM, and hypertension were more prevalent in adults with clinically diagnosed ADHD than in those without. Second, effect modification by sex was detected on the additive and/or multiplicative scales for the associations of adult ADHD with all comorbidities. Finally, ADHD remained associated with all comorbidities in adults aged 50 to 64, when all conditions were assessed from age 50 onwards.

Our findings reinforce the associations of adult ADHD with SUD, depression, bipolar disorder, and anxiety found in prior studies [5, 21, 22]. The observed prevalence estimates of SUD (35.12%), depression (42.28%), bipolar disorder (14.29%), and anxiety (44.65%) in adults with ADHD were similar to previously reported [21, 23–25]. Although the mechanisms underlying these associations are not well understood, we know from both epidemiologic and molecular genetic studies that a shared genetic predisposition might account for the co-existence of two or more psychiatric conditions [26–28]. In addition, individuals with ADHD may experience increased difficulties as the demands of life increase, which may contribute to the development of depression and anxiety [29].

Our findings about associations of clinically diagnosed ADHD with T2DM and hypertension might reflect health-risk behaviors among adult patients with comorbid ADHD. As others have noted [30], inattention, disinhibition, and disorganization associated with ADHD could make it difficult for patients to adhere to treatment regimens for metabolic disorders. ADHD-associated deficits in delayed discounting [31] and reinforcement sensitivity [32] could impair the future-oriented activities needed to promote physical health. It is also conceivable that ADHD and metabolic disorders share underlying causes [7], such as oxidative stress [33], immune dysfunction,[34] and inflammation [35]. The association between adult ADHD and hypertension is especially intriguing, as one of the leading animal models of ADHD is the spontaneously hypertensive rat [36] and two antihypertensive drugs, clonidine [37] and guanfacine [38], are efficacious for treatment of ADHD. Yet, stimulant treatment for ADHD is associated with increased systolic blood pressure [39], and it is unclear whether the association between adult ADHD and hypertension observed in the current study was mediated by stimulant treatment for ADHD. The topic deserves further investigation in a separate study. In addition, smoking and obesity are associated with ADHD [40, 41], while they also contribute to insulin resistance [42, 43], a fundamental component of T2DM etiology, and hypertension [44, 45]. This may serve as yet another explanation for the associations of ADHD with T2DM and hypertension.

In contrast to our findings, the prevalence estimates of hypertension or other cardiovascular diseases were similar between adults with ADHD and controls in a study based on U.S. health care claims [46]. The discrepancy between their results and ours could to some degree be attributed to differences in diagnostic criteria for ADHD; Diagnostic and Statistical Manual of Mental Disorders (DSM) criteria used in the U.S. are not as strict as the ICD criteria used in Sweden. Another potential explanation could be that patients with adult ADHD and their family members are overrepresented in unemployment and thus less likely to be covered by employer-sponsored health insurance [46], effectively introducing bias to the estimates in their study.

Among adults with ADHD, we found that the prevalence estimates of depression, bipolar disorder, and anxiety were higher in females than in males, whereas the prevalence estimates of SUD, T2DM, and hypertension were higher in males than in females. The top three comorbidities of adult ADHD were all psychiatric conditions, namely, SUD, depression, and anxiety, with the most common condition being anxiety in females and SUD in males. T2DM is the least common comorbidity in both females and males with ADHD. Unlike prior studies showing no evident sex differences in associations between adult ADHD and psychiatric comorbidities [9, 10], we detected effect modification by sex on the additive scale and/or multiplicative scale for the associations of adult ADHD with all psychiatric and metabolic comorbidities of interest. When sex did not act as an effect modifier on one scale, it could still do on the other. Without a more detailed pathophysiological understanding of an association, it is difficult to speculate which scale is more biologically relevant. Hence, studies examining sex differences exclusively on a multiplicative scale might overlook key information concerning sex-specific mechanisms. There is general consensus among epidemiologists that assessing additive effect modification is most appropriate for public health decision-making [19]. In the current study, while females had higher baseline prevalence of depression, bipolar disorder, and anxiety than males, adult ADHD was associated with larger absolute prevalence increase for these comorbidities in females than in males. Provided that these comorbidities could be prevented or mitigated through early detection and proper treatment of ADHD, targeting females with ADHD could increase the efficiency of prevention for these conditions. Similarly, targeting males with ADHD could increase the efficiency of prevention for SUD and hypertension. In addition, more research is needed to explore whether the sex differences in absolute prevalence, PD, and PR reflect sex-specific biological mechanisms underlying the associations.

Our findings about comorbidity of ADHD in older adults (50 to 64 years) provide a large-scale corroboration of previous findings from a few available studies on this topic. One small study of adults aged 60 to 94 years showed excess risk of depression and anxiety symptoms in those with ADHD compared to those without [13]. Another small pilot study of adults aged 60 to 85 years reported elevated rates of SUD, mood disorders and anxiety disorders in individuals with ADHD compared to controls [14]. In the current study, despite a small proportion of adults (0.29%) continuing to receive ADHD diagnosis at age 50 or older, associations with ADHD were not limited to SUD, depression, and anxiety, but also bipolar disorder, T2DM, and hypertension. Taken together, these findings highlight the need for more research in older adults, even in those older than 65 years. Given that treatments for ADHD are effective in improving ADHD symptoms, it is possible that such treatment may have a positive impact on the psychiatric and metabolic outcomes in the aging population.

It should be acknowledged that the current study was subject to certain limitations. First, ADHD was generally under-diagnosed in adults and largely overlooked in the older group (aged 45 to 64 years by the end of 2013) primarily because ADHD as a diagnostic category was rarely considered for adults in Sweden before 2002. Patients treated in primary care or failing to seek medical care were not covered by the National Patient Register. Consequently, the study could not avoid a certain amount of false negatives, giving rise to an underestimation of the associations. Second, all conditions under study were assessed via registered ICD diagnoses. Although differences in ICDs could lead to different estimates of point prevalence, patients diagnosed with these conditions tended to represent relatively severe cases and have more frequent visits to clinicians, which might induce detection bias and overestimation of the associations. Despite the first and second limitations, the true associations are still likely to be statistically significant given the magnitude of the estimates. Third, although ADHD is a persistent condition, its onset by diagnostic criteria typically precedes the onset of the comorbidities. Further, individuals who received the first diagnosis of ADHD in adulthood are likely to be those who were undiagnosed and untreated in childhood. However, due to a lack of data on outpatient specialty care before 2001 and primary care, we could not determine whether individuals with a diagnosis of ADHD in adulthood had ever been diagnosed in childhood, or deduce the extent to which the development of comorbidities was related to delay in detection and management of childhood ADHD symptoms. Neither could we infer the temporal order of the onsets of the comorbidities. The intriguing question deserves thorough investigation in future longitudinal research. Fourth, in the current study, 83.5% of all adults with an ADHD diagnosis in the National Patient Register had at least one prescription of ADHD medication. However, the Prescribed Drug Register was first introduced in July 2005, which makes it difficult to infer whether the initiation of pharmacological treatment for ADHD preceded the first diagnoses of comorbid conditions. In addition, information on non-pharmacological treatment is not available in the registers. Future research should therefore address the role of pharmacological as well as non-pharmacological treatment for ADHD using appropriate study designs.

In conclusion, the comorbidity patterns of adult ADHD underscore the severity and clinical complexity of the disorder. Clinicians should remain vigilant for a wide range of psychiatric and metabolic problems in ADHD affected adults of all ages and both sexes.

## Supporting information

S1 Table International Classification of Diseases (ICD) codes (Swedish version) for ADHD and comorbidities. (DOCX)
