# Supplementary material for: Common psychiatric and metabolic comorbidity of adult attention-deficit/hyperactivity disorder: A population-based cross-sectional study
Source: PLoS One. 2018 Sep 26;13(9):e0204516. doi: 10.1371/journal.pone.0204516 (PMC6157884; doi:10.1371/journal.pone.0204516)
Supplement: S1 Table — (DOCX) [file pone.0204516.s001.docx]

**Supporting information**

**S1 Table. International Classification of Diseases (ICD) Codes (Swedish Version) for ADHD and Comorbidities**

| **Disorder** | **ICD-8** | **ICD-9** | **ICD-10** |
| --- | --- | --- | --- |
| **ADHD** | NA | 314 | F90 |
| **Substance use disorder** | 303, 304 | 303, 304, 305 | F10 – F19 |
| **Depression** | 296.0, 300.4 | 296B, 300E, 311 | F32, F33 |
| **Bipolar disorder** | 296.1, 296.2, 296.3, 296.8 | 296A, 296C, 296D, 296E, 296W | F30, F31 |
| **Anxiety** | 300 (except 300.4) | 300 (except 300E) | F40, F41, F42, F44, F45, F48 |
| **Type 2 diabetes mellitus** | NA | NA | E11 |
| **Hypertension** | 400 – 404 | 401 – 405 | I10 – I15 |
